# Synthesis of Chitosan-Silver Nanoparticle Composite Spheres and Their Antimicrobial Activities 

**DOI:** 10.3390/polym13223990

**Published:** 2021-11-18

**Authors:** Erisna Mirda, Rinaldi Idroes, Khairan Khairan, Trina Ekawati Tallei, Muliadi Ramli, Nanda Earlia, Aga Maulana, Ghazi Mauer Idroes, Muslem Muslem, Zulkarnain Jalil

**Affiliations:** 1 Department of Chemistry, Faculty of Mathematics and Natural Sciences, Universitas Syiah Kuala, Banda Aceh 23111, Indonesia; erisnamirda@gmail.com (E.M.); muliadiramli@unsyiah.ac.id (M.R.); 2 Department of Pharmacy, Faculty of Mathematics and Natural Sciences, Universitas Syiah Kuala, Banda Aceh 23111, Indonesia; khairankhairan@unsyiah.ac.id; 3 Department of Biology, Faculty of Mathematics and Natural Sciences, Sam Ratulangi University, Manado 95115, Indonesia; trina_tallei@unsrat.ac.id; 4 General Hospital Zainoel Abidin, Banda Aceh 24415, Indonesia; nandadv09@gmail.com; 5 Department of Informatics, Faculty of Mathematics and Natural Sciences, Universitas Syiah Kuala, Banda Aceh 23111, Indonesia; aga.maulana@s1.informatika.unsyiah.ac.id; 6 Department of Chemical Engineering, Faculty of Engineering, Universitas Syiah Kuala, Banda Aceh 23111, Indonesia; idroesghazi@gmail.com; 7 Department of Chemistry, Faculty of Science and Technology, Universitas Islam Negeri Ar-Raniry, Banda Aceh 23111, Indonesia; moslem_coolam@yahoo.com; 8 Department of Physics, Faculty of Mathematics and Natural Sciences, Universitas Syiah Kuala, Banda Aceh 23111, Indonesia; zjalil@unsyiah.ac.id

**Keywords:** silver nanoparticles, chitosan, antimicrobial activity

## Abstract

Synthesis of silver nanoparticles–chitosan composite particles sphere (AgNPs-chi-spheres) has been completed and its characterization was fulfilled by UV–vis spectroscopy, Fourier transform infrared (FT-IR) spectroscopy, X-ray diffraction (XRD), scanning electron microscopy (SEM), and zetasizer nano. UV–vis spectroscopy characterization showed that AgNPs-chi-spheres gave optimum absorption at a wavelength of 410 nm. The XRD spectra showed that the structure of AgNPs-chi-spheres were crystalline and spherical. Characterization by SEM showed that AgNPs-chi-spheres, with the addition of 20% of NaOH, resulted in the lowest average particle sizes of 46.91 nm. EDX analysis also showed that AgNPs-chi-spheres, with the addition of a 20% NaOH concentration, produced particles with regular spheres, a smooth and relatively nonporous structure. The analysis using zetasizer nano showed that the zeta potential value and the polydispersity index value of the AgNPs-chi-sphere tended to increase with an increased NaOH concentration. The results of the microbial activity screening showed that the AgNP-chi-Spheres with highest concentration of NaOH, produced the highest inhibition zone diameters against *S. aureus*, *E. coli*, and *C. albicans*, with inhibition zone diameters of 19.5, 18.56, and 12.25 nm, respectively.

## 1. Introduction

Nanotechnology has been vying for the world’s attention since its rapid development in the fields of science, technology, industry, and health. Nanotechnology allows for the modification and synthesis of materials at the nanometer (nm) scale. The synthesis of nanoparticles (1–100 nm) has shown various advantages, including: minimizing material requirements, increasing activity due to increased surface area, and having unique chemical-physical properties. Past studies indicate the wide synthesis of metal nanoparticles because they have superior biological, optical, electrical, catalytic activity, and antimicrobial properties [1,2].

Silver nanoparticles have several unique features, including electronic properties, catalytic properties, optical properties [3], high surface energy, and less aggregation, in addition to several other physical and chemical properties which are not possessed by other metal nanoparticles [4]. Silver nanoparticles have also been widely used in various fields such as photocatalyst, lithography, microelectronic biosensor materials, and pharmaceuticals. It can be used as an alternative to the use of a natural product as a medicinal substance which requires raw materials from biodiversity which are starting to become less available [5,6]. Silver nanoparticle has shown good antibacterial, antifungal, and antiviral activity. [7] It is reported that silver nanoparticles could inhibit the growth of Gram-positive bacteria (*Staphylococcus aureus* and *Bacillus* sp.), Gram-negative bacteria (*Escherichia coli*, *Proteus* sp., *Pseudomonas* sp., *Serratia* sp., *Klebsiella* sp.), and pathogenic fungi (*Aspergillus niger*, *Aspergillus flavus*, *Aspergillus fumigatus*, *Candida albicans*) [5]. It is also reported that silver nanoparticles could inhibit the growth of the *C. militaris* fungus. In addition, silver nanoparticles are active against viruses such as the hepatitis B virus [8], Herpes Virus Type 1 [9], HIV (human immunodeficiency virus) [10], and influenza A H1NI virus [11]. AgNPs should provide antiviral activity against SARS-CoV-2 [12].

In this decade, the synthesis of silver nanoparticles using bio-synthetic or green synthesis methods has developed rapidly. For example, the use of materials or plant extracts as reducing agents in the synthesis of silver nanoparticles. This method has been chosen by many experts because of several advantages, including producing better antibacterial activity, as well as being inexpensive and eco-friendly [13]. There are two processes that occur in the synthesis of silver nanoparticles, namely the process of reducing Ag^+^ ions to Ag°, and the process of stabilizing the particle size. Hydrazine (N_2_H_4_), methanol (CH_3_OH), sodium borohydride (NaBH_4_), and sodium hydroxide (NaOH) can be used as reducing agents for the synthesis of silver nanoparticles. As stabilizing agent, micelles, ligands, and polymers have been widely used in past research [1,9]. The use of a polymer as a nanoparticle stabilizer is crucial to prevent the aggregation of nanoparticles, enabling the improvement of their biocompatibility properties [14]. Polymers can act as matrix materials for the growth and stability of nanoparticles [15]. The immobilization of metal nanoparticles in a polymer matrix is an effective way in reducing metal nanoparticle aggregation and increasing the biocompatibility of the metal nanoparticles. This is caused by the ability of the matrix polymer to control and stabilize the metal nanoparticle. Some commonly used polymers for synthesizing silver nanoparticles spheres are chitosan, gelatin [16], liposomes, polylactic acid [17], and polymethylmethacrylate [18], which are known to be used as a reducing agent or stabilizers of metal nanoparticles [5]. Several researchers have used polymers for the synthesis of silver nanoparticle spheres. For example, polysaccharides from microalgae [19], polystyrene [5], and chitosan [20] have successfully been used in the synthesis of silver nanoparticles. Chitosan is used as a stabilizing agent in the synthesis of sulfur nanoparticles because it has high viscosity, antimicrobial activity, biodegradable, is non-toxic, safer and eco-friendly [21,22]. In addition, structurally, chitosan has amine (-NH_2_) and hydroxyl (-OH) functional groups which act as chelating and reducing agents in the synthesis of silver nanoparticles. Chitosan is also known to function to prevent silver-nanoparticle agglomeration [23,24,25,26].

Silver-microsphere nanoparticle composites are essential for antimicrobial applications. The synthesis of silver-microsphere nanoparticle composites in the past decade has developed rapidly. It is well established that silver-microsphere nanoparticle composites have a more stable, homogeneous shape and are more effective as bactericidal and fungicidal agents [5]. For example, silver-microsphere nanoparticles synthesized using methacrylic acid as a polymer is an effective bactericidal agents for water purification [27]. Microsphere-silver nanoparticles using polymers as a stabilizer agent are also effective as antimicrobials against *Candidal* spp., *Escherichia coli, Pseudomonas* sp., *Aspergillus niger*, or *Penicillium* sp. [27,28]. Zain et al., (2014) also reported that chitosan-silver nanoparticle composites, using ascorbic acid as a reducing agent, were able to reduce the size of silver nanoparticles [29]. Akamaz et al., (2013) reported that a silver-chitosan composite, using NaOH as a reducing agent at 95 °C, was able to increase antimicrobial activity against *Escherichia coli*, *Acinetobacter baumannii*, *Staphylococcus aureus*, *Enterococcus faecalis*, *Pseudomonas aeruginosa*, and *Streptococcus pneumonia* [30].

In this research, we approached the synthesis of microsphere-silver nanoparticles using chitosan as a stabilizer and sodium hydroxide (NaOH) as a reducing agent. The concentration of NaOH used in this study varied from the lowest concentration (20%) to the highest (50%). The silver-chitosan nanoparticles microsphere composite (AgNPs-chi-spheres) was characterized using UV-Vis spectroscopy, FT-IR spectroscopy, scanning electron microscopy (SEM), X-ray diffraction (XRD) and zetasizer nano. In this study, the antimicrobial activity of AgNPs-chi-spheres against *Staphylococcus aureus*, *Escherichia coli* and *Candida albicans* was also determined. 

## 2. Methods

### 2.1. Materials

The materials used in the study were chitosan (molecular weight: 150,000, 1.5% *w*/*v*), AgNO_3_, glucose, and sodium hydroxide (NaOH), which were purchased from Sigma-Aldrich Co. (St. Louis, MO, USA). All received materials were used immediately without further purification.

### 2.2. Synthesis of Chitosan-Spheres (Chi-Spheres)

The chitosan-spheres were synthesized following the procedure of Wang et al., (2015) with some modifications [5]. A chitosan solution was prepared by dissolving 0.2 grams of chitosan into 10 mL of 1% (*v*/*v*) acetic acid (CH_3_COOH) and stirring using a magnetic stirrer at 250 rpm for 3 h at room temperature until the chitosan was dissolved entirely. Then the solution was dripped into 25 mL of 20%, 30%, 40%, and 50% NaOH solution using a syringe pump. After 15 min, the solution was then centrifuged at 6000 rpm for 10 min. The resulting supernatant was then discarded, while the residual spheres produced were then washed with 30 mL of double-distilled water (ddH_2_O) to remove the alkaline solution from NaOH; the washing process with ddH_2_O was carried out twice [5]. The resulting spheres were then dried at 40 °C for 1 h to obtain chitosan-spheres (chi-spheres) [31].

### 2.3. Synthesis of Silver Nanoparticles–Chitosan Composite Spheres (AgNPs-Chi-Spheres)

A chitosan solution was made by dissolving 0.2 grams of chitosan into 10 mL of 1% (*v*/*v*) acetic acid (CH_3_COOH) and stirring at 250 rpm for 3 h at room with a magnetic stirrer until the chitosan was completely dissolved. To produce a complex solution of AgNP-chitosan, 10 mL of 2% AgNO_3_ was gradually added into the chitosan solution while stirring using a magnetic stirrer at a speed of 200 rpm for 24 h. Then the solution was dripped into 25 mL of 20%, 30%, 40%, and 50% NaOH solutions, respectively, using a syringe pump. After 15 min, when the color changed to brown, indicating the formation of AgNP-chitosan, the solution was centrifuged at 6000 rpm for 10 min. The supernatant was then discarded. The resulting residual spheres were washed with 30 mL of double-distilled water (ddH_2_O) to remove the alkaline solution. The washing process with ddH_2_O was carried out twice [5]. The resulting spheres were then dried at 40 °C for 1 h to obtain silver nanoparticles–chitosan composite spheres (AgNPs-chi-spheres) [31].

### 2.4. Characterizations of AgNPs-Chi-Spheres

#### 2.4.1. UV–Vis Spectral Analysis

In the UV–Vis spectral analysis, the silver nanoparticles solution was prepared by adding 20 μL CH_3_COOH solution and 1 mL dd-H_2_O to 10 silver nanoparticles–chitosan composite spheres (Ag-chi-spheres), and then vortexed for 3–5 min. The absorption of AgNPs-Chi-spheres was observed using a UV-Vis spectrophotometer (Shimadzu, UV 2500, Shimadzu Suzhou Instruments Mfg. Co, Ltd, 145 Huashan Road, New District Suzhou, Suzhou, Jiangsu, China) at a wavelength of 300 to 700 nm.

#### 2.4.2. FT-IR Analysis

AgNPs-chi-spheres were grounded with KBr pellets and analyzed using FT-IR spectroscopy (Cary 630 FTIR spectrometer, Agilent Technologies, 5301 Stevens Creek Blud, Santa Clara, CA, USA). The FT-IR spectrum analysis of AgNPs-chi-spheres was carried out at a 500–4500 cm^−1^ wavelength.

#### 2.4.3. XRD Analysis

An XRD characterization (XRD-6000 Shimadzu, 7102 Riverwood Drive, Columbia, MD, USA) was conducted using Cu and Kα as radiation sources, and Ni as a filter with an energy of 30 kV/30 mA. For XRD analysis, AgNPs-chi-spheres samples were placed on glass substrates. The XRD pattern was taken at room temperature with its Tesla angle (2θ) of 10° ≤ 2θ ≤ 70°.

#### 2.4.4. SEM-EDX Analysis

The micromorphology and size of AgNPs-chi-spheres were analyzed using a Scanning Electron Microscope/Energy Dispersive Using X-Ray (SEM-EDX, Sem-eds Carl Zeiss-Bruker EVO MA 10, Carl Zeiss Microscopy, One North Broadway, White Plains, NY, USA) Quanta FEI 450 SEM machine at an energy of 15 kV with 5000× and 10,000× magnification.

#### 2.4.5. Zetasizer Nano Analysis

The characterization of the zeta potential value (mV) and the polydispersity index (PI) of AgNPs-chi-spheres were analyzed using a zetasizer nano (Horiba SZ-100, Horiba Mfg. Co. Ltd, Kyoto, Japan), using the dynamic light scattering method, with a sample temperature holder of 24.8 °C, a conductivity value of 0.093 mS/cm and a voltage of 3.9 V.W.

### 2.5. Antimicrobial Activity

Screening for antibacterial activity was carried out on *S. aureus* (gram-positive), *E. coli* (gram-negative), and *Candida albican* (a common opportunistic fungal organism capable of causing skin infections) using the agar well diffusion method. The microbial suspensions were made according to McFarland standards. Each 1 mL of microbial suspension was poured into a petri dish. Following that, 25 mL of Mueller Hinton Agar (MHA) media was added to screen for antibacterial activity and Sabouraud Dextrose Agar (SDA) media was added to screen for antifungal activity. Then, the media were homogenized evenly. Six steel rings with a diameter of 6 mm were placed on the media for each petri dish and the media were allowed to harden. After that, the six steel rings were removed so as to form wells in the media. The silver nanoparticles–chitosan solution was prepared by adding 20 μL CH3COOH solution and 1 mL dd-H_2_O to 10 ml Ag-chi-spheres, and then vortex for 3–5 min. Subsequently, 50 μL Ag-chi-spheres solutions was added to each well. In this assay, an antibiotic of ciprofloxacin and ketoconazole was used as positive controls for antibacterial and antifungal activities, respectively. 

The samples were subsequently incubated at 37 °C for 24 h. The clear zone formed was then measured, which indicated the presence of antimicrobial activity [5]. 

## 3. Results and Discussion

### 3.1. Synthesis of Chitosan-Spheres (Chi-Spheres)

**Figure 1:**
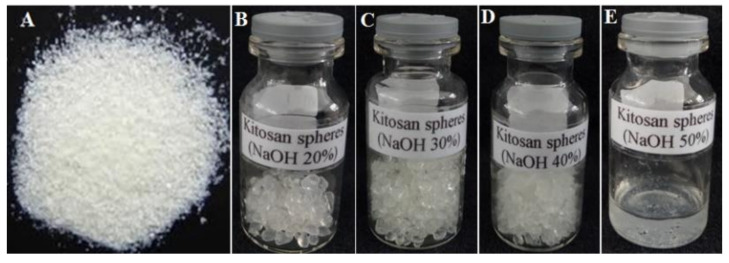
(**A**) Chitosan (chi) and optimization product of synthesizing chitosan-spheres (chi-spheres) using (**B**) 20%; (**C**) 30%; (**D**) 40%; (**E**) 50% NaOH.

Figure 1 shows the formation of chi-spheres at various concentrations of NaOH. Chi-spheres made using 20% NaOH, produced better spheres with better spherical shape and homogeneity than those made using 30% and 40% NaOH. Interestingly, chitosan made with 50% NaOH did not form chi-spheres; this is presumably because the high NaOH concentration resulted in higher alkaline conditions, thus inhibiting the formation of chi-spheres (spheres thickening due to high alkaline conditions) (Figure 1E). This study also determined the percentage yield of chi-spheres, as shown in Table 1.

Table 1 shows that an addition 30% NaOH resulted in a higher percentage yield of 1.30, followed by an addition of 20% NaOH with a percentage yield of 1.17. Meanwhile, higher concentrations of NaOH, namely at 40% and 50%, resulted in a lower percentage yield of 0.8 and 0.5, respectively.

### 3.2. Synthesis of Silver Nanoparticles–Chitosan Composite Spheres (AgNPs-Chi-Spheres)

**Figure 2:**
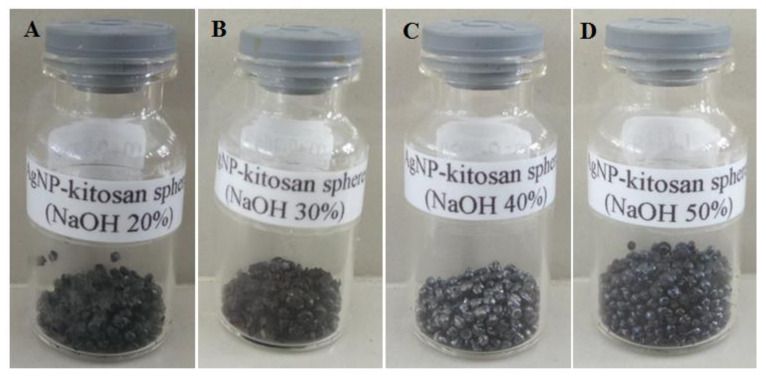
Products of AgNPs-chi-spheres. (**A**) AgNPs-chi-spheres 1 (NaOH 20%); (**B**) AgNPs-chi-spheres 2 (NaOH 30%); (**C**) AgNPs-chi-spheres 3 (NaOH 40%); (**D**) AgNPs-chi-spheres 4 (NaOH 50%).

Chitosan solution added with 2% AgNO_3_ solution, and several variations of NaOH concentration, forms AgNPs-chi-spheres, characterized by a color change to brown or blackish brown. Figure 2 shows the AgNPs-chi-spheres synthesis. The results show that AgNPs-chi-spheres at made 40% and 50% NaOH produce better AgNPs-chi-spheres that are more homogeneous, compact, and shiny (Figure 2C,D) compared to AgNPs-chi-spheres made with 20% and 30% NaOH (Figure 2A,B). These results indicate that the concentration of NaOH plays an important role in forming the surface of AgNPs-chi-spheres.

This study determined the percent yield of synthesized AgNPs-chi-spheres. AgNPs-chi-spheres, added to a 50% NaOH, produced a higher percentage yield of 0.55% compared to the AgNPs-chi-spheres which were added to 20%, 30%, and 50% NaOH (Table 2). The results also show that increasing the concentration of NaOH as a reducing agent tends to produce a higher percentage yield.

### 3.3. UV–Vis Spectral Analysis

**Figure 3:**
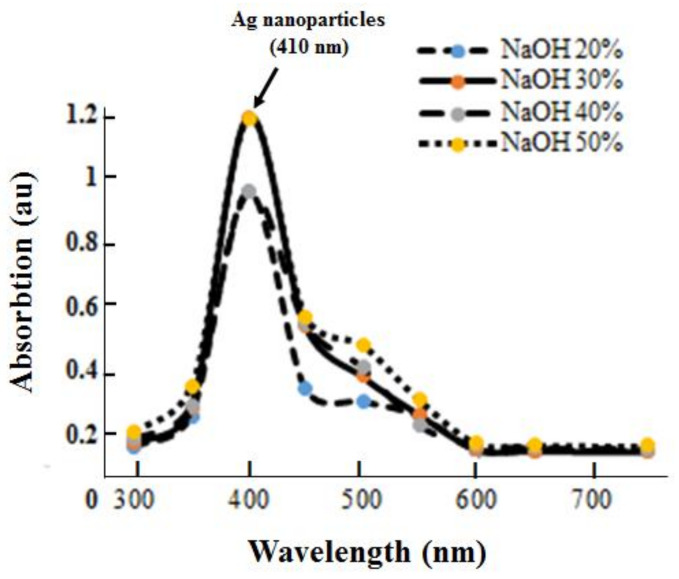
UV–Vis absorbance spectra of the synthesized silver–chitosan composite spheres at various concentrations of NaOH solution.

Figure 3 shows the UV–Vis absorption spectra of AgNPs-chi-spheres at various concentrations of NaOH. The results show optimum absorption at a 410 nm wavelength when AgNPs-chi-spheres were synthesized at various NaOH concentrations. This result is in line with research from [5], which states that silver nanoparticles absorb at a wavelength of approximately 410 nm. Figure 3 also shows no change in peak at a wavelength of 410 nm at the various NaOH concentrations. This result indicates that the addition of NaOH does not significantly affect the diameter of the synthesized silver nanoparticles [5,11].

### 3.4. FT-IR Analysis

**Figure 4:**
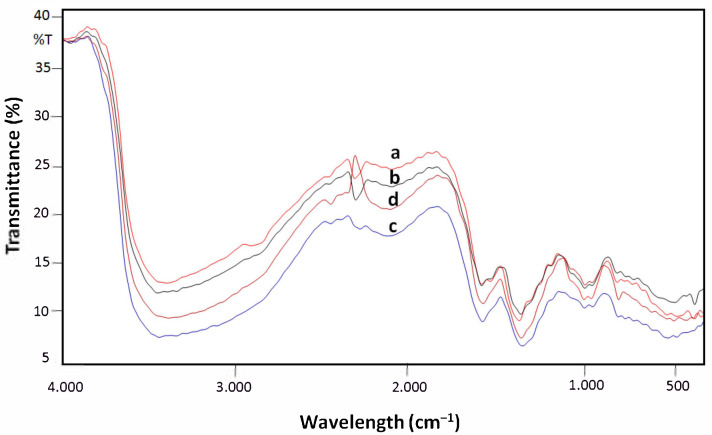
Characterization of the FT-IR spectra of AgNPs-chi-spheres. FT-IR AgNPs-chi-spheres. a. AgNPs-chi-spheres with NaOH 20%; b. AgNPs-chi-spheres with NaOH 30%; c. AgNPs-chi-spheres with NaOH 40%; d. AgNPs-chi-spheres with NaOH 50%.

Figure 4 shows the characterization of the FT-IR spectrum of AgNPs-chi-spheres with various concentrations of NaOH added to the silver nanoparticles synthesis process. The FT-IR spectrum analysis shows that AgNPs-chi-spheres with various concentrations of NaOH have similar FT-IR spectrum characteristics.

The absorption between the wavelengths of 3404.5 cm^−1^ and 3466.23 cm^−1^ is the N–H stretching of the amino group. The absorption at the wavelength of 2937.31 cm^−1^ is the C–H stretching thought to come from lipids. The absorption at the wavelength of 1649.21 cm^−1^ and 1601.95 cm^−1^ is the C–N bond of the amide compound. The absorption at wavelengths of 1426.42 cm^−1^ and 1419.67 cm^−1^ is from the (–CH_2_) group. The absorption at a wavelength of 1259.12 cm^−1^ is the C–O bond from the carboxyl group. The presence of N–H and C–N absorption seems to be due to the interaction between the amino groups and the metallic surface of silver nanoparticles (AgNPs), where the amino groups act as capping agents for the stabilization of silver nanoparticles [32,33].

### 3.5. XRD Analysis

**Figure 5:**
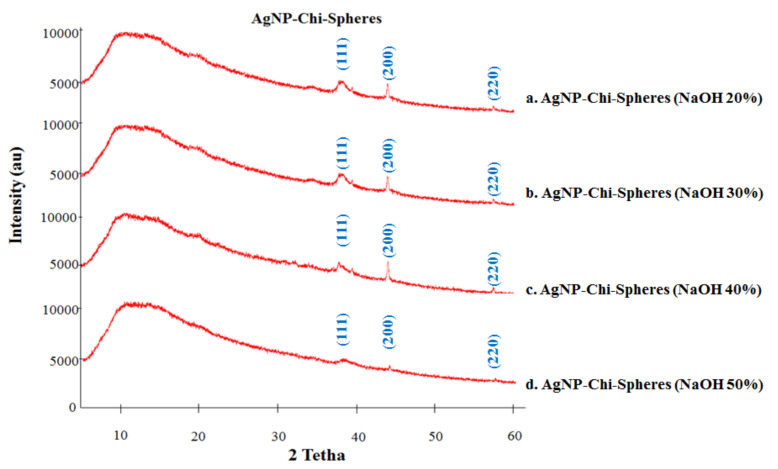
Characterization of AgNP-chi-spheres by XRD.

Figure 5 shows the XRD pattern of AgNP-chi-spheres. The XRD analysis showed that the numbers of Bragg reflections with 2θ values of 38.21°, 43.80°, and 57.48° correspond to (111), (200), and (220) respectively (Figure 5). The two characteristics of silver nanoparticles (111), (200), and (220) are consistent with those found in the Joint Committee on Powder Diffraction Standards (JCPDS) database. These indicate that the resulting nanoparticles are silver nanoparticles, as the positions and relative intensities of all the diffraction peaks are consistent with the crystalline pattern of silver. This is in line with the research conducted previously [5,7,34], that the XRD diffraction pattern characteristic for silver nanoparticles is (111), (200), (220), and (311). Furthermore, the chitosan used to synthesize silver nanoparticles–chitosan composite spheres did not cause the formation of silver oxides [34]. The results also show that the resulting spectrum tends to be wider (broad); this might be caused by the chitosan polymer [35].

### 3.6. SEM-EDX Analysis

The characterization from SEM showed that AgNPs-chi-spheres, with the addition of 20% NaOH concentration, gave the lowest average particle size with diameter of 46.91 nm. The average diameter of AgNPs-chi-spheres at various concentrations of NaOH additions can be seen in Table 3.

**Figure 6:**
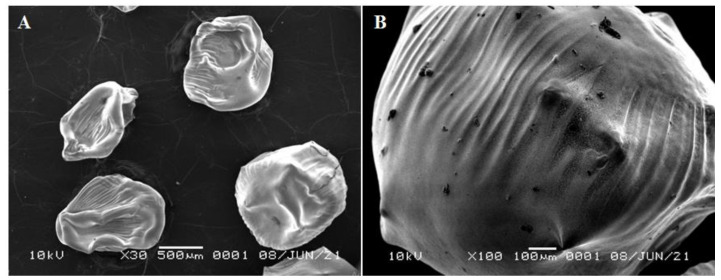
SEM spectrum of AgNP-chi-spheres using 20% NaOH, (**A**) 30×; (**B**) 100×.

Figure 6 shows the fabrication of AgNPs-chi-spheres using 20%, which resulted in the smallest nanoparticles diameter. Figure 6A shows a graph of the surface morphology of the silver nanoparticle composite spheres. Figure 6B shows a zoomed-in surface morphology of AgNPs-chi-spheres with a 20% NaOH concentration. Previous research reported that AgNPs-chi-spheres have a porous surface structure [5,36]. In our research, AgNPs-chi-spheres have regular spheres with a smooth structure and are relatively nonporous.

**Figure 7:**
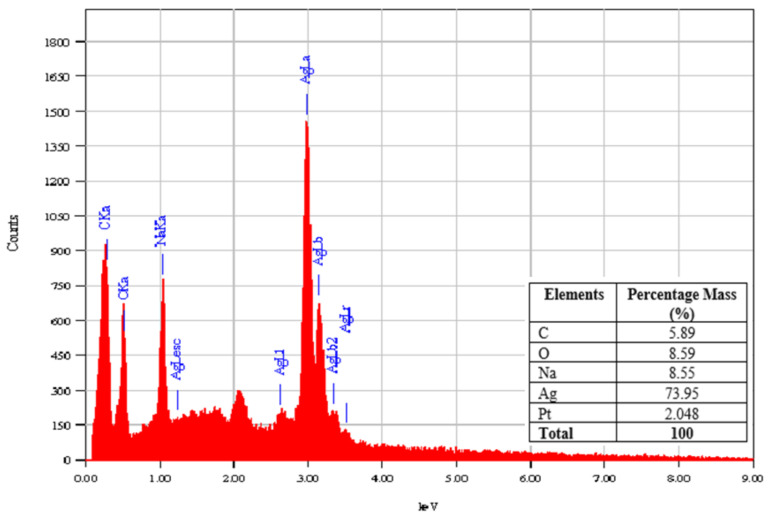
EDX spectrum of AgNP-chi-spheres using 20% NaOH.

Figure 7 shows the EDX spectrum of an AgNPs-chi-sphere made using 20% NaOH. The EDX spectrum shows that the mass percentage of silver nanoparticles produced in AgNPs-chi-sphere synthesis, using 20% NaOH, is 73.95% (Table 4). These results indicate that chitosan and NaOH can function as stabilizing and reducing agents for silver nanoparticles.

### 3.7. Zetasizer Nano Analysis

The zeta potential, a parameter of electric charge between colloidal particles, is the accumulated charge amount on the particle’s surface. The higher the value of the potential substance, the more it will prevent flocculation/colloidal merging from small to large. Particles with a low zeta potential value allow particles to attract each other and flocculate. The smaller the zeta potential, the thinner this layer and the more likely particles will coalesce [37]. A high zeta potential will produce a very stable colloid [38]. Table 4 shows the zetasizer nano analysis of the silver nanoparticles–chitosan composite spheres (AgNP-chi-spheres) with several concentrations of NaOH. 

The results show that the zeta potential value of silver nanoparticles–chitosan composite spheres increased with an increasing NaOH concentration. However, when added to 50% NaOH, the zeta potential value decreased by 36.0 mV. This indicates that increasing the NaOH concentration decreases the stability of silver nanoparticles. As shown in Table 4, AgNP-chi-spheres exhibited a high zeta potential value at various NaOH concentrations. It has been stated in the literature that nanoparticles with a zeta potential higher than +30 mV, or lower than −30 mV, are considered to be very stable in the dispersion medium.

The PdI value is used to determine the homogeneity of nanoparticles; the smaller the obtained PdI value, the more homogeneous the nanoparticles are. Nanoparticles with a PI value greater than 0.7 indicate that the resulting nanoparticles have an extensive size distribution. Meanwhile, a PI with a value of 0.3 or below indicates that the particle population is relatively homogeneous [39,40]. Table 4 shows that the PdI values of AgNP-chi-spheres at various concentrations of NaOH range from 0.1 to 0.3; this indicates that the homogeneity of the resulting particles tends to be more homogeneous [41,42]. Table 4 also shows that the PdI of silver nanoparticles increased in line with the NaOH concentration. This indicates that the addition of NaOH concentration increases the distribution of particles in the solution.

### 3.8. Antimicrobial Activity

Bacterial and fungal infections are still a significant problem in the health sectors. Therefore, developing novel antibiotics with good biocompatibility properties without resistance is crucial. Silver nanoparticles are promising candidates to be developed as antimicrobials due to their intrinsic properties and excellent thermal stability, with low toxicity to mammalian cells and tissues [43,44]. The development of silver-based antimicrobial nanocomposite materials might be advantageous, for example, by using various natural reductants such as chitosan, dextran, sodium citrate, ascorbic acid, and other biomolecular reducing agents, such as polyketides, flavones, and alkaloids. The antimicrobial activity (AgNP-Chi-Spheres) was determined in this work [5,45].

**Figure 8:**
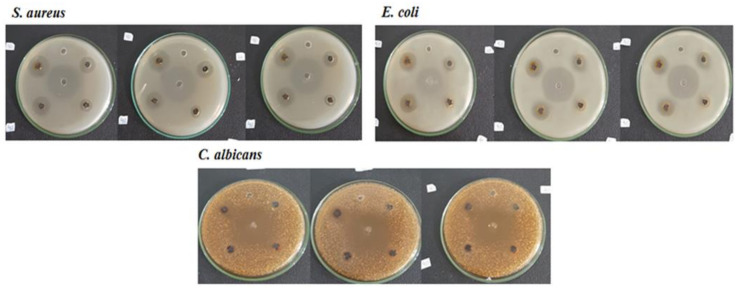
Antimicrobial activity of AgNP-chi-spheres against *S. aureus*, *E. coli*, and *C. albicans* after 24 h of incubation time.

Figure 8 shows the antimicrobial activity of AgNP-chi-spheres against *S. aureus*, *E. coli*, and *C. albicans* fungus. The results show that the greater the concentration of NaOH added to the AgNP-chi-spheres, the higher the antimicrobial activity.

Table 5 shows the inhibition zone diameter produced by AgNP-chi-spheres. The results showed that AgNP-chi-spheres added with 50% NaOH produced the highest inhibition zone diameters against *S. aureus*, *E. coli*, and *C. albicans*, with inhibition zone diameters of 19.5 nm, 18.56 nm, and 12.25 nm, respectively. These results indicate that AgNP-chi-spheres have broad antimicrobial spectrum.

## 4. Conclusions

The synthesis of silver nanoparticles using a chitosan stabilizing agent and NaOH as a reducing agent with concentrations of 20% to 50%, produces a complex form of AgNPs-chi-spheres. Characterization using UV–Vis spectroscopy showed that AgNPs-chi-spheres presented optimum absorption at a wavelength of 410 nm. XRD analysis showed that AgNPs-chi-spheres added with 50% NaOH, produced a lower intensity spectrum. Characterization using SEM, showed that AgNPs-chi-spheres added to a 20% NaOH produced the smallest average particle size of 46.91 nm. Analysis using SEM-EDX also showed that AgNPs-chi-spheres at added with 20% NaOH produced particles with regular spheres, a smooth structure, and were relatively nonporous with a 73.95% silver mass percentage. The zetasizer nano analysis showed that the zeta potential and the PdI values of AgNPs-chi-spheres tended to increase with the increase of the NaOH concentration. The microbial activity assay showed that AgNP-chi-spheres added with 50% NaOH produced the highest inhibition zone diameters against *S. aureus*, *E. coli*, and *C. albicans* with inhibition zone diameters of 19.5 nm, 18.56 nm, and 12.25 nm, respectively.

## Figures and Tables

**Table 1:** Percent yield of chi-spheres using various concentrations of NaOH. The preparation of chi-spheres was performed in duplicates.

NaOH [%]	Reactants (grams)	Products (grams)	Percentage Yield (%)
20	1.455 ± 0.067	127.96 ± 0.520	1.17 ± 0.057
30	3.238 ± 0.057	247.92 ± 1.430	1.30 ± 0.015
40	1.955 ± 0.060	247.92 ± 0.198	0.80 ± 0.024
50	1.150 ± 0.070	247.92 ± 0.050	0.50 ± 0.028

**Table 2:** Percentage yield of AgNPs-chi-spheres at various concentrations of NaOH. The preparation of AgNPs-chi-spheres were performed in duplicates.

Product	NaOH Concentration [%]	Reactants (gram)	Product (gram ± SD)	Yield (% ± SD)
A	20	167.46	0.875 ± 0.007	0.52 ± 0.005
B	30	167.46	0.749 ± 0.065	0.47 ± 0.020
C	40	167.46	0.916 ± 0.009	0.54 ± 0.001
D	50	167.46	0.930 ± 0.014	0.55 ± 0.012

**Table 3:** Diameter size of AgNPs-chi-spheres.

Reducing Agent	Average Diameter ± SD (nm)
NaOH 20%	46.91 ± 1.50
NaOH 30%	58.36 ± 1.70
NaOH 40%	73.92 ± 1.80
NaOH 50%	77.81 ± 1.80

**Table 4:** Zetasizer nano analysis of AgNP-chi-spheres. The zeta potential and PI values were performed in duplicates.

No.	Reducing Agent	Zeta Potensial (mV ± SD)	Polydisperity Index (PdI ± SD)
1.	NaOH 20%	33.6 ± 0.70	0.171 ± 0.05
2.	NaOH 30%	47.2 ± 0.07	0.256 ± 0.01
3.	NaOH 40%	52.3 ± 0.70	0.249 ± 0.03
4.	NaOH 50%	36.0 ± 1.27	0.323 ± 0.02

**Table 5:** Antimicrobial activity of AgNP-chi-spheres after 24 h of incubation time. The inhibition zone diameter was performed in triplicates.

No.	Tested Microorganisms	AgNP-Chi-Spheres (in % NaOH)	Inhibition Zone Diameter (mm ± SD)
1.	*S. aureus*	Amoxicillin 0.50% (C^+^)	30.22 ± 0.020
		20	15.40 ± 0.015
		30	11.51 ± 0.015
		40	16.55 ± 0.015
		50	19.51 ± 0.015
2.	*E. coli*	Ciprofloxasin 0.25% (C^+^)	23.55 ± 0.020
		20	12.86 ± 0.015
		30	10.68 ± 0.015
		40	16.05 ± 0.017
		50	18.56 ± 0.017
3.	*C. albicans*	Ketoconazole 0.20% (C^+^)	24.87 ± 0.020
		20	10.27 ± 0.026
		30	10.13 ± 0.020
		40	11.16 ± 0.015
		50	12.25 ± 0.017

## Data Availability

The data presented in this study are available on request from the corresponding author.
